# Comprehensive In Silico Functional Prediction Analysis of CDKL5 by Single Amino Acid Substitution in the Catalytic Domain

**DOI:** 10.3390/ijms232012281

**Published:** 2022-10-14

**Authors:** Yuri Yoshimura, Atsushi Morii, Yuuki Fujino, Marina Nagase, Arisa Kitano, Shiho Ueno, Kyoka Takeuchi, Riko Yamashita, Tetsuya Inazu

**Affiliations:** 1 Graduate School of Pharmaceutical Sciences, Ritsumeikan University, Kusatsu 525-8577, Shiga, Japan; 2 Department of Pharmacy, College of Pharmaceutical Sciences, Ritsumeikan University, Kusatsu 525-8577, Shiga, Japan

**Keywords:** CDKL5, in silico prediction analysis, PolyPhen-2, PROVEAN, SIFT, single amino acid substitutions

## Abstract

Cyclin-dependent kinase-like 5 (CDKL5) is a serine/threonine protein kinase whose pathological mutations cause CDKL5 deficiency disorder. Most missense mutations are concentrated in the catalytic domain. Therefore, anticipating whether mutations in this region affect CDKL5 function is informative for clinical diagnosis. This study comprehensively predicted the pathogenicity of all 5700 missense substitutions in the catalytic domain of CDKL5 using in silico analysis and evaluating their accuracy. Each missense substitution was evaluated as “pathogenic” or “benign”. In silico tools PolyPhen-2 HumDiv mode/HumVar mode, PROVEAN, and SIFT were selected individually or in combination with one another to determine their performance using 36 previously reported mutations as a reference. Substitutions predicted as pathogenic were over 88.0% accurate using each of the three tools. The best performance score (accuracy, 97.2%; sensitivity, 100%; specificity, 66.7%; and Matthew’s correlation coefficient (MCC), 0.804) was achieved by combining PolyPhen-2 HumDiv, PolyPhen-2 HumVar, and PROVEAN. This provided comprehensive information that could accurately predict the pathogenicity of the disease, which might be used as an aid for clinical diagnosis.

## 1. Introduction

Rett syndrome (RTT) is an X-linked neurodevelopmental disorder first reported in 1966 by Andreas Rett, a pediatrician in Vienna [1]. Major causative genes for RTT include methyl-CpG-binding protein 2 (MECP2), cyclin-dependent kinase-like 5 (CDKL5, also known as STK9), and forkhead box protein G1 (FOXG1) [2,3,4]. CDKL5 (STK9) was first reported as a gene encoding a novel serine/threonine protein kinase mapped to X chromosome p22 [5]. CDKL5 mutations are involved in X-linked infantile spasms and mental retardation [3,6,7]. Mutations in MECP2 cause most cases of classic RTT, whereas mutations in CDKL5 and FOXG1 cause atypical RTT [8,9,10].

Recently, the disease associated with atypical RTT containing a CDKL5 mutation has been distinguished from RTT and is called “CDKL5 deficiency disorder (CDD)”. CDD affects approximately 1 in 40,000 live births [11]. 

The N-terminus of CDKL5 has a catalytic domain and the C-terminus has a unique regulatory domain including two nuclear localization signal (NLS) sequences and a nuclear export signal (NES) [12]. CDD is driven by the loss of CDKL5 kinase activity. There are many CDKL5 mutations identified in patients with CDD including missense, nonsense, indel, and splice site [3,6,7,13,14,15,16]. Therefore, these mutations lead to changes in authentic CDKL5 kinase activity or protein structure such as truncated protein or fusion protein. The mutations change their enzyme activity and intracellular localization then finally lead to causing disease.

To date, over 265 pathogenic variants within CDKL5 have been reported [17]. Approximately 50% of these variants are point mutations, while missense mutations are most commonly identified within the catalytic domain (38%) [17]. Missense mutations are predicted to change CDKL5 activity and lead to disease onset. Indeed, several missense mutations are characterized as loss-of-function mutations [13,18]. Thus, detecting CDKL5 activity is important for identifying disease-causative mutation(s). 

The recent development of next-generation sequencing (NGS), including exome sequencing, is commonly available for precise molecular diagnosis in many fields such as epileptic encephalopathies and Rett syndrome [19,20]. Causative gene mutations for the diseases were clarified; however, variants of uncertain significance (VUS) were occasionally determined. The relationship between genotype and phenotype and the significance of these variants was not clarified. 

This study virtually changed authentic catalytic domain amino acids in CDKL5 to 19 different amino acids to comprehensively predict functional changes using in silico tools. The vast majority of amino acid variants in the catalytic domain became pathogenic. This approach identified the pathogenicity of the disease which might aid clinical diagnosis.

## 2. Results

### 2.1. The CDKL5 Catalytic Domain Is Highly Conserved

**Figure 1:**
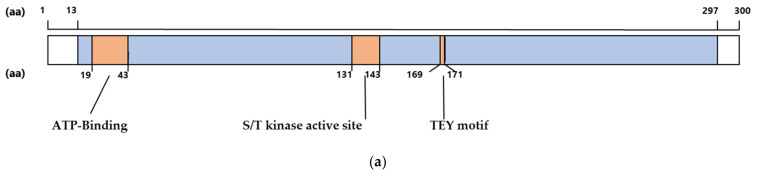

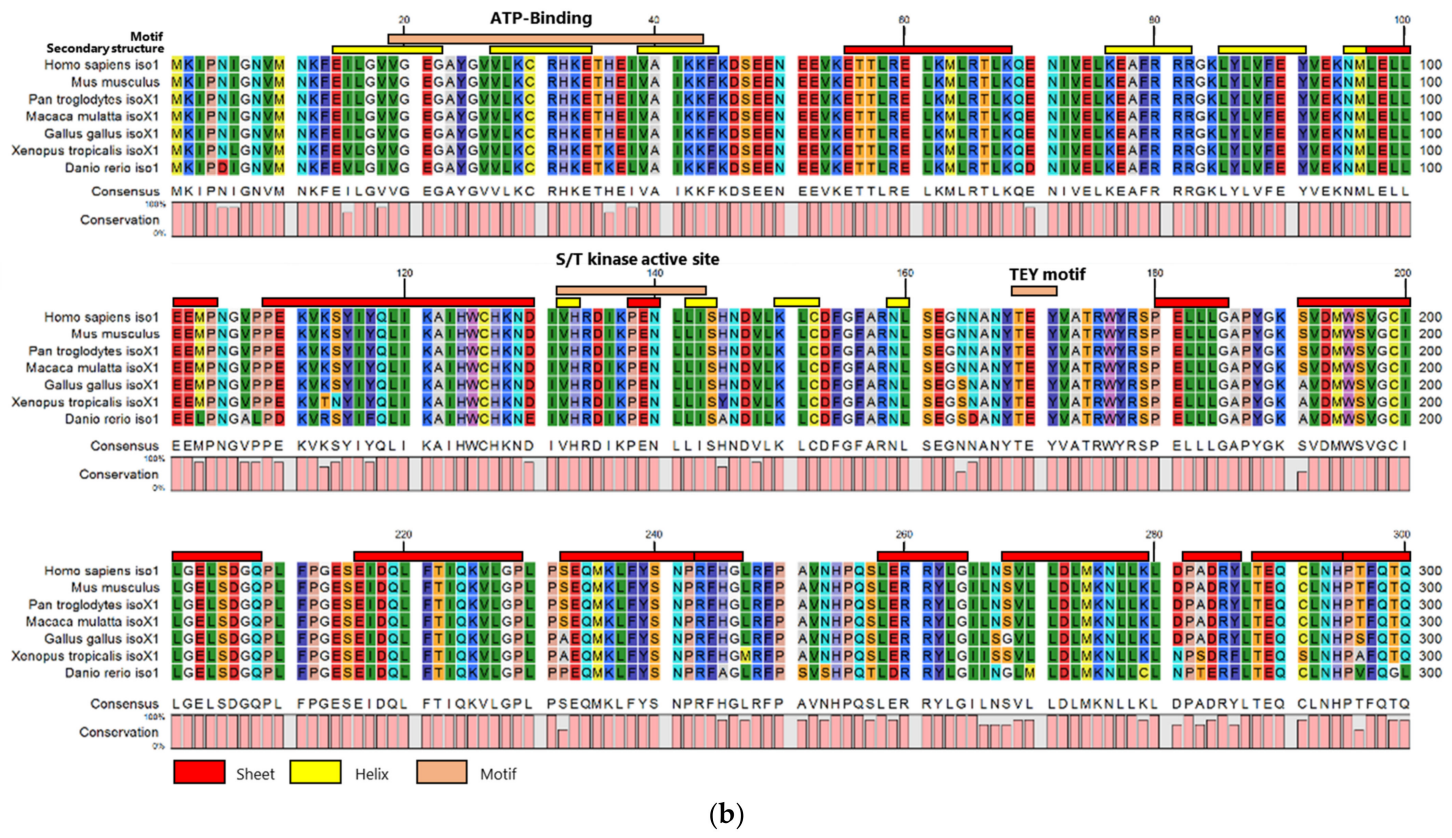
(**a**) Schematic illustration of human CDKL5 primary structure showing amino acids 1 to 300. The catalytic domain is colored blue. ATP-binding site, S/T kinase active site, and the TEY sequence are shown in orange. aa; amino acids (**b**) CDKL5 amino acid sequences of Mus musculus, Pan troglodytes, Macaca mulatta, Gallus, Xenopus tropicalis, and Danio rerio were aligned with CDKL5 from Homo sapiens. Orange lines indicate the ATP-binding site, S/T kinase active site, and TEY motif. Red and yellow lines indicate β-sheets and α-helices, respectively. The graph below the sequences indicates the match rate of each position. Identical amino acid positions in two-thirds of CDKL5s are shaded light gray.

The conservation of amino acid sequences in the catalytic domain was initially analyzed to determine whether it is important in CDKL5 (Figure 1a). Several animal CDKL5 sequences obtained from NCBI (https://www.ncbi.nlm.nih.gov, accessed on 14 November 2021) were aligned (Figure 1b). The similarity between humans and other species was 100% for mice, chimpanzees, and rhesus monkeys; 98.0% for chickens; 94.7% for platanna; and 87.7% for zebrafish. Therefore, the catalytic domain of CDKL5 is highly conserved. In particular, the ATP-binding site, S/T kinase active site, and TEY motif are highly conserved (Figure 1a) [21]. 

### 2.2. Mutations in the CDKL5 Catalytic Domain Were Predicted to Affect Its Function

The pathogenicity of various missense variants in the CDKL5 catalytic domain was predicted using four in silico prediction tools: PolyPhen-2 HumDiv/HumVar, PROVEAN, and SIFT (Figures S1–S4). A summary of the prediction results was shown in Table 1. 

PolyPhen-2 scores from 5700 mutations showed that 95.8% of mutations in HumDiv mode and 92.3% of mutations in HumVar mode are “probably damaging” and “possibly damaging”, while 4.2% in HumDiv and 7.7% in HumVar are “benign” mutations (Table 1, Figures S1 and S2). 

PROVEAN scores judged 82.6% of mutations as “deleterious” and 17.4% of mutations as “neutral” (Table 1, Figure S3). Notably, mutations between amino acids 1 and 9 were judged as “neutral”, indicating that the mutation is likely to have no effect. 

The SIFT score judged 88.0% of mutations as “deleterious” and 12.0% of mutations as “tolerated” (Table 1, Figure S4). 

### 2.3. Validation of ClinVar Data Demonstrated the Validity of the In Silico Analysis

The accuracy of the analysis results was verified using the already published data that has the 36 CDKL5 mutations, including 33 pathogenic mutations and 3 benign mutations from ClinVar data (Table S1). These data appeared in ClinVar and have been reviewed by their expert panel. The criteria used for validating a combination of tools followed previous work by Leong et al. [22] (Table 2). The results of the validation are shown in Table 3.

The score of 15 of prediction tools or combinations was evaluated based on Matthew’s correlation coefficient (MCC) score (Table 3). The score by 10 prediction tools was above 0.5, indicating that these analyses classified the mutations with high accuracy (Table 3). The largest MCC of 0.804 was obtained with the (i) PolyPhen-2 (HumVar) and PROVEAN analysis; and (ii) PolyPhen-2 (HumDiv), PolyPhen-2 (HumVar), and PROVEAN analysis. In particular, combining multiple tools scored an accuracy of 97.2%, sensitivity of 100%, specificity of 66.7%, and MCC of 0.804 (Table 3). Therefore, validation using multiple tools is effective for the pathogenicity prediction of CDKL5 mutations.

**Figure 2:**
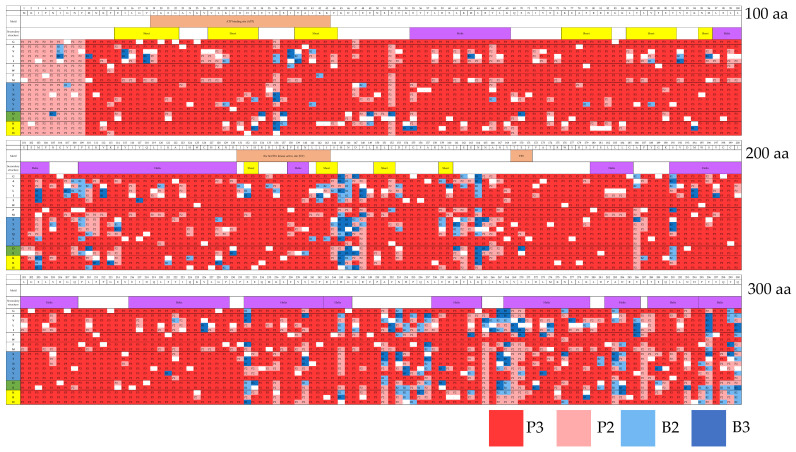
Heatmap of integrated in silico prediction data from PolyPhen-2 (HumDiv), PolyPhen-2 (HumVar), and PROVEAN analysis. The criteria for judgment were indicated in Table 2. There were 94.0% pathogenic mutations and 6.0% benign mutations out of 5700 mutations. Amino acid sequences are shown at the top of the table, where amino acids were divided into non-polar and polar amino acids, polar amino acids, those without acidity (blue), as well as acidic amino acids (green), and basic amino acids (yellow). Wild-type (WT) amino acids such as M1M, K2K, I3I, … were indicated “.” And colored in white. The mutation judgments P3, P2, B2, and B3 are shown in the smallest square. aa; amino acids.

Integrated results of in silico prediction data were obtained by combining PolyPhen-2 (HumDiv), PolyPhen-2 (HumVar), and PROVEAN tools (Figure 2). We unified the words pathogenic (=damaging, =deleterious) and benign (=neutral, =tolerated) to avoid terminology confusion. The evaluation results were classified into pathogenic groups (P3 and P2) and benign groups (B2 and B3) (Table 4). There was a total of 94.0% pathogenic mutations (P3, 79.6%; P2, 14.4%) and 6.0% benign mutations (B2, 3.2%; B3, 2.8%) (Figure 2).

We compared the relationship between amino acid conservations and functional prediction concerning Figure 2. Among various species, the amino acid conservations in critical sites are 98.3% in ATP-binding site, 100% in S/T kinase active site and TEY motif, with 96.3% and 92.3% in the non-critical sites in the catalytic region (amino acids; aa No. 144–168, No. 257–297), respectively.

However, regarding the functional prediction, pathogenic 3 (P3) and pathogenic 2 (P2) in critical regions were 87.6% and 8.0%, respectively, in ATP-binding site; 89.9% and 5.3%, respectively, in S/T kinase active site; and 96.5% and 1.8%, respectively, in TEY motif. Whereas P3 in the non-critical sites was 66.6% and 67.4%, P2 in these regions was 18.7% and 21.7%, respectively. 

Benign 3 (B3) and benign 2 (B2) in critical regions were 2.11% and 2.3 % respectively in ATP-binding site, 0.81% and 4.0% respectively in S/T kinase active site, 1.81% and 2.7% respectively in TEY motif. Whereas B3 in the non-critical sites was 5.78% and 7.37%, B2 in those regions was 6.5% and 5.9%, respectively. As a result of statistical processing, the sum of P3 and P2 in the critical region was significant compared to non-critical regions and B3 and B2 in critical regions were significant compared to non-critical regions (95% confidence interval).

Finally, we evaluated previous data [13,18,23,24,25] using the optimal combined in silico strategy as verification (Figure 2). Twenty mutations previously evaluated for activity in vitro but not included within ClinVar classification (except three cases) were compared to in silico predictions (Tables S2 and S3). Nineteen of the previously reported mutations had significantly decreased in vitro CDKL5 kinase activity or changed the subcellular localization of the protein. Meanwhile, the H36R mutation with significantly increased in vitro activity was predicted to be “benign” by all tools used in the current in silico analysis. The remaining inactivating mutations are as follows: PolyPhen-2 HumDiv, 17 mutations were predicted as “probably damaging” and 1 as “possibly damaging”; PolyPhen-2 HumVar, 15 mutations were predicted as “probably damaging” and 3 as “possibly damaging”; PROVEAN, 18 mutations were predicted as “deleterious”. Overall, 18 mutations judged to be damaging (=deleterious) by all three tools are in good agreement with in vitro analysis. The two benign mutations using in silico analysis are in relatively good agreement with in vitro analysis. However, opposing results were obtained for H145Y by in silico analysis compared with in vitro results. These results suggested that the in silico prediction is unable to correctly judge “damaging” (=deleterious) mutations determined by in vitro analysis. Nevertheless, 95% of in silico and in vitro analyses coincided suggesting that the in silico analysis used in this study is highly accurate.

## 3. Discussion

The amino acid sequences in the catalytic domain of CDKL5 were highly conserved among various species. A total of 5700 virtual missense variants in the catalytic domain of CDKL5 were examined by comprehensive in silico functional prediction analysis to select the best-performing combination of in silico prediction tools.

The prediction performance of four in silico tools/modes was compared, including PolyPhen-2 HumDiv, PolyPhen-2 HumVar, PROVEAN, and SIFT. The combination of two PolyPhen-2 modes and PROVEAN produced the highest accuracy (97.2%) and sensitivity. The combination of three tools/modes produced an MCC of 0.864 which was larger than 0.5; this indicated that mutations were classified with high accuracy. However, the MCC score of SIFT was −0.051, which is close to −1; this indicated that the classification of mutations was incorrectly predicted. In the present case in SIFT, since there were only three benign mutations for evaluation, it was considered that one misclassification had a large impact on the MCC value, resulting in a negative score. Furthermore, a previous study has reported that the specificity of SIFT is significantly lower than its sensitivity [26]. Hence, it could be said that SIFT is characterized by a high probability of judging benign as pathogenic and this property may have influenced the results.

Concerning specificity, we obtained 66.7% specificity by this method which was not a high score. Therefore, we might need to use an alternative method for future analysis.

Combined tool analysis for ClinVar data had an accuracy of 97.2% and 95% using another dataset based on an in vitro experimental study. This suggests that the prediction might be a useful and important guide for clinical diagnosis.

Regarding the result in Figure 2, we compared the relationship between amino acid conservations and functional prediction. We found that there was a significant correlation between them.

The main limitation of this study is the small number of benign single nucleotide variants (SNVs) reported in the CDKL5 gene compared to pathogenic SNVs.

In fact, only 36 the mutations, which seems to be a low number, were used for the calculations of accuracy, sensitivity, specificity and MCC. Therefore, a small number of benign SNVs is problematic [22]. Information on benign SNVs is important for calculating specificity in in silico tool analysis. Hence, this method may be unable to correctly predict benign SNVs. We consider that it is important to diagnose benign mutations appropriately based on the analysis data we have generated and other data. Therefore, it will be important to determine if the predicted benign mutations identified in this study correlate with known benign mutations by developing an in vitro assay system. Furthermore, the ability to determine functional abnormalities, such as CDKL5 activity, would make this tool more predictive of SNVs. This may be important to improve accuracy.

The second limitation was the inability to directly obtain VUS data. A total of 53 VUSs were found in the catalytic domain of CDKL5 from ClinVar. Then, most variants, such as F13I, G20S, and T35P, were judged as “probably damaging” and “deleterious” in this study, suggesting that the evaluation is pathogenic (P3 level). On the other hand, there are some variants judged as benign in this study: N267S and S268N (B3 level). Therefore, the judgment of VUSs should be made not only by in silico analysis but also by clinical significance and in vitro functional analysis as ACMG/AMP guideline recommended [27]. Recently, the evolutionary model of variant effect (EVE) made predictions for 3219 disease-causing genes (including CDKL5) by relying on the evolutionary distribution of sequence variation across organisms [28]. This powerful approach is also available for in silico functional prediction of CDKL5 and also discriminates VUS. Therefore, our analysis and EVE may compensate for each other’s limitations.

Functional characterization of each CDKL5 mutation found in clinical practice is extremely burdensome for clinicians who often rely on in silico predictive tools for diagnosis. This study confirms that comprehensive clinical judgment (including analysis of these tools) is important for determining pathogenicity. It is important to accumulate data on pathogenic mutations (especially benign mutations) through clinical studies and to corroborate these data with in vitro analysis to further improve diagnostic accuracy. However, it is not easy to collect data from CDD because it is a rare disease. Therefore, the collection of data from large databases such as UK Biobank and gnomAD is needed to obtain benign variant data. Otherwise, well-established in vitro functional studies will predict the precise effect on protein function [27]. Easy, reproducible, high throughput, and comprehensive in vitro functional analysis of the disease is important for precise molecular diagnosis.

## 4. Materials and Methods

### 4.1. Sequence Data

The amino acid sequences of CDKL5 from various species were obtained from NCBI (https://www.ncbi.nlm.nih.gov, accessed on 14 November 2021). The following gene sequences of CDKL5, cyclin-dependent kinase-like 5 isoform 1 were obtained from different species: *Homo sapiens*: NM_003159.3, *Mus musculus*: NM_001024624.2, *Pan troglodytes*: XM_024353159.1, *Macaca mulatta*: XM_028842229.1, *Gallus*: XM_040647995.2, *Xenopus tropicalis*: XM_031896705.1, *Danio rerio*: NM_001145768.1.

### 4.2. Bioinformatics Analysis

The following in silico prediction tools (including previously described methodology and algorithms) were used to analyze the single nucleotide variants (SNVs) of CDKL5: PolyPhen-2 (HumVar and HumDiv) [29], PROVEAN [30], and SIFT [31,32]. The analysis range is from amino acids 1 to 300 which is the catalytic domain of CDKL5 [21]. Each authentic human amino acid was replaced with 19 different amino acids for a total of 5700 mutations (19 × 300 amino acid mutations). The effects of the mutations were scored.

PolyPhen-2 (http://genetics.bwh.harvard.edu/pph2/, last accessed on 5 July 2022) is a tool that scores and predicts whether the missense mutation is in a structurally important site of the protein [29]. In addition, the model calculates the degree of harm using machine learning methods based on the above scores using a known mutation dataset as a teacher data set. There are two modes in PolyPhen-2: HumDiv and HumVar. HumVar is well suited for diagnosing Mendelian diseases by distinguishing mutations with significant consequences from other human mutations, including minor ones. On the other hand, HumDiv is useful for assessing rare alleles at loci that may be involved in complex phenotypes, for high-density mapping of regions identified by genome-wide association studies, and for natural selection analysis of sequence data, in which even mildly lethal alleles are treated as harmful.

This study considered each mode as an independent tool, then used both modes together. HumDiv is the default setting and is suited for rare alleles, while HumVar is suited for Mendelian disease diagnostics. The magnitude of the effects was assessed by PolyPhen-2 scores ranging from 0.0 (benign) to 1.0 (damaging). The mutation was classified as “probably damaging” if its score was above 0.957 in Hum Div and 0.909 in HumVar as “possibly damaging”. A score below 0.450 in Hum Div and 0.447 in HumVar was classified as “benign” [29,33]. SNVs assigned as “probably damaging” or “possibly damaging” were classified as “pathogenic” for downstream analysis.

PROVEAN (https://www.jcvi.org/research/provean, last accessed on 17 December 2021) and SIFT (http://siftdna.org/www/Extended_SIFT_chr_coords_submit.html, last accessed on 5 July 2022) quantify the degree of abnormality resulting from mutations in amino acids based on the degree of conservation of the mutation site from the sequence alignment. These tools rely on evolutionary sequence conservation information and do not consider protein structure information [30,31,32]. Therefore, SIFT has been primarily applied to human polymorphisms; it can be applied to any organism because it is based on the principles of protein evolution. PROVEAN provides a generalized approach to predict the functional effects of protein sequence variations including single or multiple amino acid substitutions, and in-frame insertions and deletions. This alignment-based score measures the change in sequence similarity of a query sequence to a protein sequence homolog before and after the introduction of an amino acid variation to the query sequence.

PROVEAN looks at the conservation of amino acid sequences across species, amino acid substitution frequency, and chemical properties. It calculates delta scores for possible substitutions to 20 amino acids, with lower delta scores when mutations have adverse effects. The delta score also takes into account the alignment of neighboring regions.

The PROVEAN score sets a cutoff value of −2.5 and mutations with a score below the criteria are defined as “deleterious” [30]. “Deleterious” and “neutral” were hereafter referred to as “pathogenic” and “benign”, respectively, for consistency and to avoid confusion.

SIFT is a tool that uses sequence homology from multiple sequence alignments to predict the pathogenicity of mutations. The default settings were used; SIFT scores each variant on a scale from 1.00 (tolerated) to 0.00 (deleterious). A mutation with a score of 0.05 or above was classified as “tolerated”, and anything below 0.05 was classified as “deleterious” [31,32]. “Deleterious” and “tolerated” were hereafter referred to as “pathogenic” and “benign” for consistency and to avoid confusion.

All analyses were performed on the software, and scores were double-checked by different researchers. PolyPhen-2 (v2.2.3) and SIFT were last accessed on 5 July 2022, while PROVEAN v1.1.3 was last used on 17 December 2021.

### 4.3. Assessment of In Silico Prediction Analysis Data Using Reference Data from ClinVar

The accuracy of the output analysis outcome was evaluated by checking whether the pathogenicity was consistent with that of previously reported CDKL5 mutations.

Reference data included pathogenic and benign CDKL5 mutations obtained from the ClinVar database (https://www.ncbi.nlm.nih.gov/clinvar/ which was last accessed on 5 August 2022). ClinVar provides clinically significant data for pathogenic, benign, or likely benign mutations. Data exhibiting high-confidence variants were selected by the following criteria: (1) review status of criteria has been provided, (2) at least one submitter and/or reviewed by an expert panel. ClinVar criteria containing 33 pathogenic mutations and 3 benign and likely benign mutations were extracted from the original pool of 44 pathogenic mutations and 3 benign mutations. In silico predictions of each selected mutation were used to calculate the performance of each tool or any combinations of each tool using the following methods.

The method for determining the combination of multiple tools was performed according to the criteria stated by Leong et al. [22] (Table 2). These results were classified as true positive (TP, correct prediction of damaging mutation), true negative (TN, correct prediction of benign mutation), false positive (FP, false prediction of benign mutation), and false negative (FN, false prediction of damaging mutation); these four categories were used to determine the accuracy, sensitivity (true positive rate), and specificity (true negative rate) of each in silico tool.

Accuracy is the percentage of true results (true positives or true negatives) in the population and was calculated as Equation (1) [34]

(1) $$\left[\frac{\mathrm{TP}+\mathrm{TN}}{\left(\mathrm{TP}+\mathrm{TN}+\mathrm{FP}+\mathrm{FN}\right)}\right]\times 100$$

Sensitivity is defined as the probability of identifying a true mutation and was calculated as Equation (2) [35]

(2) $$\left[\frac{\mathrm{TP}}{\left(\mathrm{TP}+\mathrm{FN}\right)}\right]\times 100$$

Specificity was defined as the probability of identifying a true negative mutation and was calculated as Equation (3) [35]

(3) $$\left[\frac{\mathrm{TN}}{\left(\mathrm{TN}+\mathrm{FP}\right)}\right]\times 100$$

Matthew’s Correlation Coefficient (MCC) was calculated as Equation (4) [35]

(4) $$\frac{\left(\mathrm{TP}\times \mathrm{TN}\right)-\left(\mathrm{FP}\times \mathrm{FN}\right)}{\sqrt{\left(\mathrm{TP}+\mathrm{FP}\right)\times \left(\mathrm{TP}+\mathrm{FN}\right)\times \left(\mathrm{TN}+\mathrm{FP}\right)\times \left(\mathrm{TN}+\mathrm{FN}\right)}}\;$$

It was calculated for a single tool or for each combination [36]. MCC measures how well the prediction correlates with the actual target value, with scores ranging from +1 (always correct) to −1 (always wrong), with 0 indicating a completely random prediction [36]. The MCC score was 0.5 when 75% of cases were correctly predicted; therefore, a measured MCC score of 0.5 or higher was considered acceptable [36].

Predictions were determined by the number of pathogenic/benign predictions (Table 4). The “P” of P2 and P3 indicates pathogenic and “B” of B2 and B3 indicates benign. P3 refers to mutations with three pathogenic predictions by PolyPhen-2 HumDiv, HumVar, and PROVEAN. P2 refers to mutations with two pathogenic predictions by two of PolyPhen-2 HumDiv, HumVar, and PROVEAN. B2 and B3 were assigned in the same way.

### 4.4. Statistical Processing between Critical Region and Non-Critical Region

The number of P3, P2, B2, B3 predictions in critical region and non-critical region are counted. As critical region, ATP-binding site (19–43 amino acids = aa), S/T kinase active site (131–143aa), and TEY motif (169–171aa) are selected. As non-critical site, region between 144–168aa (between S/T kinase active site and TEY motif) and region between 257–297aa are selected to represent. Comparisons between two groups were made using test of proportion. Statistical significance was set at 95% confidence interval.

## 5. Conclusions

A comprehensive in silico functional analysis of CDKL5 showed that the combination of PolyPhen-2 HumDiv, PolyPhen-2 HumVar, and PROVEAN tools gave the best performance for predicting the severity of mutations. The obtained data will be useful for clinical diagnosis.

## Figures and Tables

**Table 1:** Summary of Prediction tool results.

Prediction Tool		Benign	Pathogenic	Total
PolyPhen-2 HumDiv	prediction	Benign	Probably damaging/ Possibly damaging	
	frequency	241	5459	5700
	rate	(4.2%)	(95.8%)	(100%)
PolyPhen-2 HumVar	prediction	Benign	Probably damaging/ Possibly damaging	
	frequency	440	5260	5700
	rate	(7.7%)	(92.3%)	(100%)
PROVEAN	prediction	Neutral	Deleterious	
	frequency	990	4710	5700
	rate	(17.4%)	(82.6%)	(100%)
SIFT	prediction	Tolerated	Deleterious	
	frequency	686	5014	5700
	rate	(12.0%)	(88.0%)	(100%)

**Table 2:** Conditions for single nucleotide variant (SNV) data output from 2 to 3 and all in silico missense prediction tools are considered to be either benign or damaging.

Number of In Silico Prediction Tools	SNV Considered as Benign	SNV Considered as Damaging
2 tools	Unanimously neutral/tolerated/benign	Unanimously probably damaging/ possibly damaging/deleterious
		One output is probably damaging/ possibly damaging/deleterious
3 tools	Unanimously neutral/tolerated/benign	Unanimously probably damaging/ possibly damaging/deleterious
	Two outputs are neutral/tolerated/benign	Two outputs are probably damaging/ possibly damaging/deleterious
4 tools	Unanimously neutral/tolerated/benign	Unanimously probably damaging/ possibly damaging/deleterious
	Three outputs are neutral/tolerated/benign	Two or more outputs are probably damaging/ possibly damaging/deleterious

**Table 3:** The accuracy, sensitivity, specificity, and MCC (Matthew’s correlation coefficient) scores of all combinations of in silico prediction tools for CDKL5 variants.

		CDKL5			
	Prediction Tools or Combinations	Accuracy	Sensitivity	Specificity	MCC
		(%)	(%)	(%)	
	PolyPhen-2 HumDiv	94.4	100	33.3	0.561
	PolyPhen-2 HumVar	94.4	97.0	66.7	0.636
	PROVEAN	94.4	97.0	66.7	0.636
	SIFT	88.9	97.0	0.0	−0.051
	PolyPhen-2 HumDiv & PolyPhen2 HumDiv	94.4	100	33.3	0.561
	PolyPhen-2 HumDiv & PROVEAN	94.4	100	33.3	0.561
	PolyPhen-2 HumDiv & SIFT	91.7	100	0.0	―
	PolyPhen-2 HumVar & PROVEAN	97.2	100	66.7	**0.804**
	PolyPhen-2 HumVar & SIFT	91.7	100	0.0	―
	PROVEAN & SIFT	88.9	97.0	0.0	−0.051
★	PolyPhen-2 HumDiv & PolyPhen-2 HumVar & PROVEAN	97.2	100	66.7	**0.804**
	PolyPhen-2 HumDiv & PolyPhen-2 HumVar & SIFT	94.4	100	33.3	0.561
	PolyPhen-2 HumDiv & PROVEAN & SIFT	91.7	97	33.3	0.366
	PolyPhen-2 HumVar & PROVEAN & SIFT	94.4	97	66.7	0.636
	PolyPhen-2 HumDiv & PolyPhen-2 HumVar & PROVEAN & SIFT	94.4	100	33.3	0.561

Number of the previously reported CDKL5 mutations, Pathogenic mutations; n = 33, Benign mutations; n = 3, Highest MCCs are shown in bold. Red star is used as group of tools to determine pathogenic or benign in Figure 2.

**Table 4:** Prediction methods for Figure 2.

Number of in silico prediction as neutral/tolerated/benign	0	1	2	3
Number of in silico prediction as probably damaging/possibly damaging/deleterious	3	2	1	0
Total prediction	P3	P2	B2	B3

P3, P2, B2, and B3 were differentiated by the number of pathogenic/benign predictions by PolyPhen-2 HumDiv/HumVar and PROVEAN.

## Data Availability

Not applicable.

## Supplementary Materials

The following supporting information can be downloaded at: https://www.mdpi.com/article/10.3390/ijms232012281/s1.
