# Supplementary material for: Comprehensive In Silico Functional Prediction Analysis of CDKL5 by Single Amino Acid Substitution in the Catalytic Domain
Source: Int J Mol Sci. 2022 Oct 14;23(20):12281. doi: 10.3390/ijms232012281 (PMC9603577; doi:10.3390/ijms232012281)
Supplement: Supplementary file 1 [file ijms-23-12281-s001.zip › ijms-1934913-supplementary.pdf]

International Journal of
Molecular Sciences

MDPI

Supplementary information

Table S1. Pathogenic and benign mutations used for Table 3 calculations for each *in silico* prediction tool.

Pathogenic mutations

AA1	Pos.	AA2	PolyPhen-2_Div	PolyPhen-2_Var	PROVEAN	SIFT
F	13	S	Probably damaging	Probably damaging	Deleterious	Deleterious
G	20	V	Probably damaging	Probably damaging	Deleterious	Deleterious
G	25	R	Probably damaging	Probably damaging	Deleterious	Deleterious
A	40	V	Probably damaging	Probably damaging	Deleterious	Deleterious
K	42	R	Possibly damaging	Possibly damaging	Deleterious	Deleterious
R	65	P	Probably damaging	Probably damaging	Deleterious	Deleterious
N	71	D	Probably damaging	Probably damaging	Deleterious	Deleterious
I	72	T	Probably damaging	Probably damaging	Deleterious	Deleterious
V	73	M	Probably damaging	Probably damaging	Deleterious	Deleterious
L	119	R	Probably damaging	Probably damaging	Deleterious	Deleterious
A	122	T	Probably damaging	Probably damaging	Deleterious	Deleterious
H	127	R	Probably damaging	Probably damaging	Deleterious	Deleterious
P	138	L	Probably damaging	Probably damaging	Deleterious	Deleterious
P	138	A	Probably damaging	Probably damaging	Deleterious	Deleterious
N	140	S	Probably damaging	Possibly damaging	Deleterious	Deleterious
K	150	R	Possibly damaging	Benign	Deleterious	Deleterious
V	172	I	Probably damaging	Probably damaging	Neutral	Tolerated
W	176	C	Probably damaging	Probably damaging	Deleterious	Deleterious
R	178	P	Probably damaging	Probably damaging	Deleterious	Deleterious
R	178	G	Probably damaging	Probably damaging	Deleterious	Deleterious
R	178	Q	Probably damaging	Probably damaging	Deleterious	Deleterious
R	178	W	Probably damaging	Probably damaging	Deleterious	Deleterious
E	181	A	Probably damaging	Probably damaging	Deleterious	Deleterious
D	193	N	Probably damaging	Probably damaging	Deleterious	Deleterious
D	193	A	Probably damaging	Probably damaging	Deleterious	Deleterious
W	195	G	Probably damaging	Probably damaging	Deleterious	Deleterious
S	196	L	Probably damaging	Probably damaging	Deleterious	Deleterious

G	202	W	Probably damaging	Probably damaging	Deleterious	Deleterious
G	207	V	Probably damaging	Probably damaging	Deleterious	Deleterious
G	213	E	Probably damaging	Probably damaging	Deleterious	Deleterious
L	220	P	Probably damaging	Probably damaging	Deleterious	Deleterious
L	277	S	Probably damaging	Probably damaging	Deleterious	Deleterious
R	285	K	Probably damaging	Probably damaging	Deleterious	Deleterious
Benign mutations						
AA1	Pos.	AA2	PolyPhen-2_Div	PolyPhen-2_Var	PROVEAN	SIFT
P	242	T	Probably damaging	Probably damaging	Deleterious	Deleterious
H	254	R	Possibly damaging	Benign	Neutral	Deleterious
T	296	A	Benign	Benign	Neutral	Deleterious

Both mutation data were obtained from ClinVar.

Table S2. Summary of functionally characterized CDKL5 variants *in vitro*.

mutations	% activity of Phos-Tag SDS PAGE	other *in vitro* assay	Assessment of *in vitro* assay	References
WT	100		Benign	
G20R	19		Pathogenic	[23]
G20D	19		Pathogenic	[23]
E21G	17		Pathogenic	[23]
G22V	20		Pathogenic	[23]
R31G	12		Pathogenic	[23]
T35I	14		Pathogenic	[23]
H36R	119		Benign	[23]
A40V	15	miss localization	Pathogenic	[23], [24]
K42R (ATP-binding site)	10	loss of activity	Pathogenic	[18], [23]
I72N	8		Pathogenic	[23]
G83V	13		Pathogenic	[23]
H145Y	36		Pathogenic	[23]
C152F		loss of activity	Pathogenic	[18], [25]
R175S		low auto phosphorylation, unable modification of MeCP2	Pathogenic	[18], [25]
Y177C	11	loss of activity	Pathogenic	[13], [23]
R178P	22		Pathogenic	[23]
P180L		loss of activity	Pathogenic	[25]
Q219P	17		Pathogenic	[23]
L220P	23	miss localization	Pathogenic	[23], [24]
C291Y	10		Pathogenic	[23]

The reason for the decision is shown in parentheses. "Definition of in vitro assay" indicates the result of judging the activity of each mutation by combining the % activity of Phos-tag SDS-PAGE and the results of other in vitro assays.

Table S3. Evaluation of CDKL5 mutations with known *in vitro* activity by *in silico* analysis.

mutations	PolyPhen-2 HumDiv	PolyPhen-2 HumVar	PROVEAN	SIFT	Assessment of *in silico* assay
WT					
G20R	Probably damaging	Probably damaging	Deleterious	Deleterious	P3
G20D	Probably damaging	Possibly damaging	Deleterious	Deleterious	P3
E21G	Probably damaging	Probably damaging	Deleterious	Deleterious	P3
G22V	Probably damaging	Probably damaging	Deleterious	Deleterious	P3
R31G	Probably damaging	Probably damaging	Deleterious	Deleterious	P3
T35I	Probably damaging	Probably damaging	Deleterious	Deleterious	P3
H36R	Benign	Benign	Neutral	Tolerated	B3
A40V	Probably damaging	Probably damaging	Deleterious	Deleterious	P3
K42R (ATP-binding site)	Possibly damaging	Possibly damaging	Deleterious	Deleterious	P3
I72N	Probably damaging	Probably damaging	Deleterious	Deleterious	P3
G83V	Probably damaging	Probably damaging	Deleterious	Deleterious	P3
H145Y	Benign	Benign	Neutral	Deleterious	B3
C152F	Probably damaging	Possibly damaging	Deleterious	Deleterious	P3
R175S	Probably damaging	Probably damaging	Deleterious	Deleterious	P3
Y177C	Probably damaging	Probably damaging	Deleterious	Deleterious	P3
R178P	Probably damaging	Probably damaging	Deleterious	Deleterious	P3
P180L	Probably damaging	Probably damaging	Deleterious	Deleterious	P3
Q219P	Probably damaging	Probably damaging	Deleterious	Deleterious	P3
L220P	Probably damaging	Probably damaging	Deleterious	Deleterious	P3
C291Y	Probably damaging	Probably damaging	Deleterious	Deleterious	P3

The results of each *in silico* prediction tool were evaluated for known mutations, and the relationship between *in silico* and *in vitro* data were evaluated. "Definition of *in silico* assay" is derived from the results stated in Table 4.

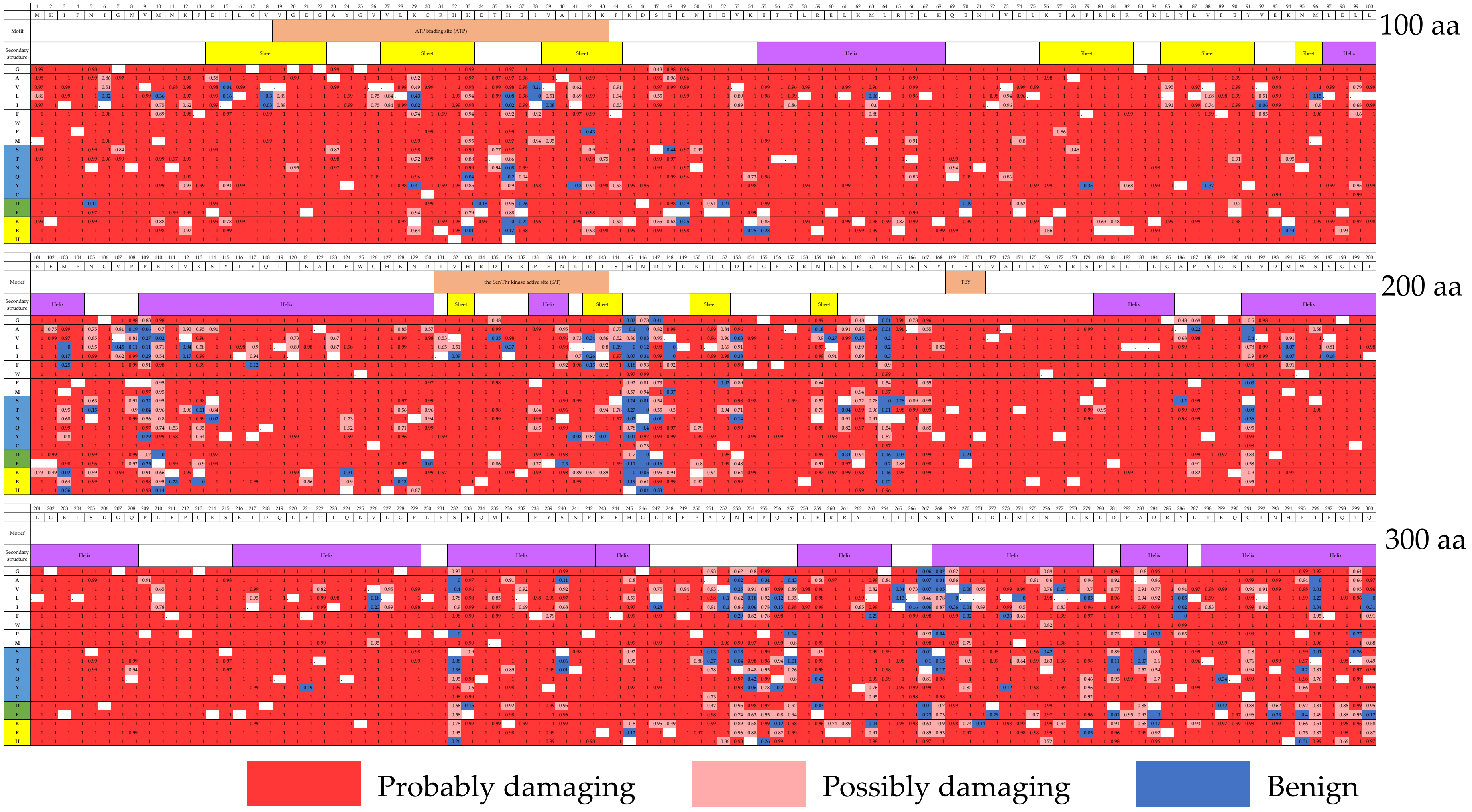

Figure S1. PolyPhen-2 HumDiv prediction heatmap. PolyPhen-2 HumDiv scores are indicated for each mutation and colored by PolyPhen-2 HumDiv prediction; those above 0.455 and 0.957 were highlighted in pink and red, respectively; those below 0.449 were highlighted in blue. Amino acid sequences are shown on the top of the table, where amino acids were categorized as non-polar and polar, neutral (blue), acidic (green), and basic (yellow). Wild type (WT) amino acids WT such as M1M, K2K, I3I, …was indicated "." and colored in white. The mutation score (0.000~1.000) is within the smallest square. aa; amino acids

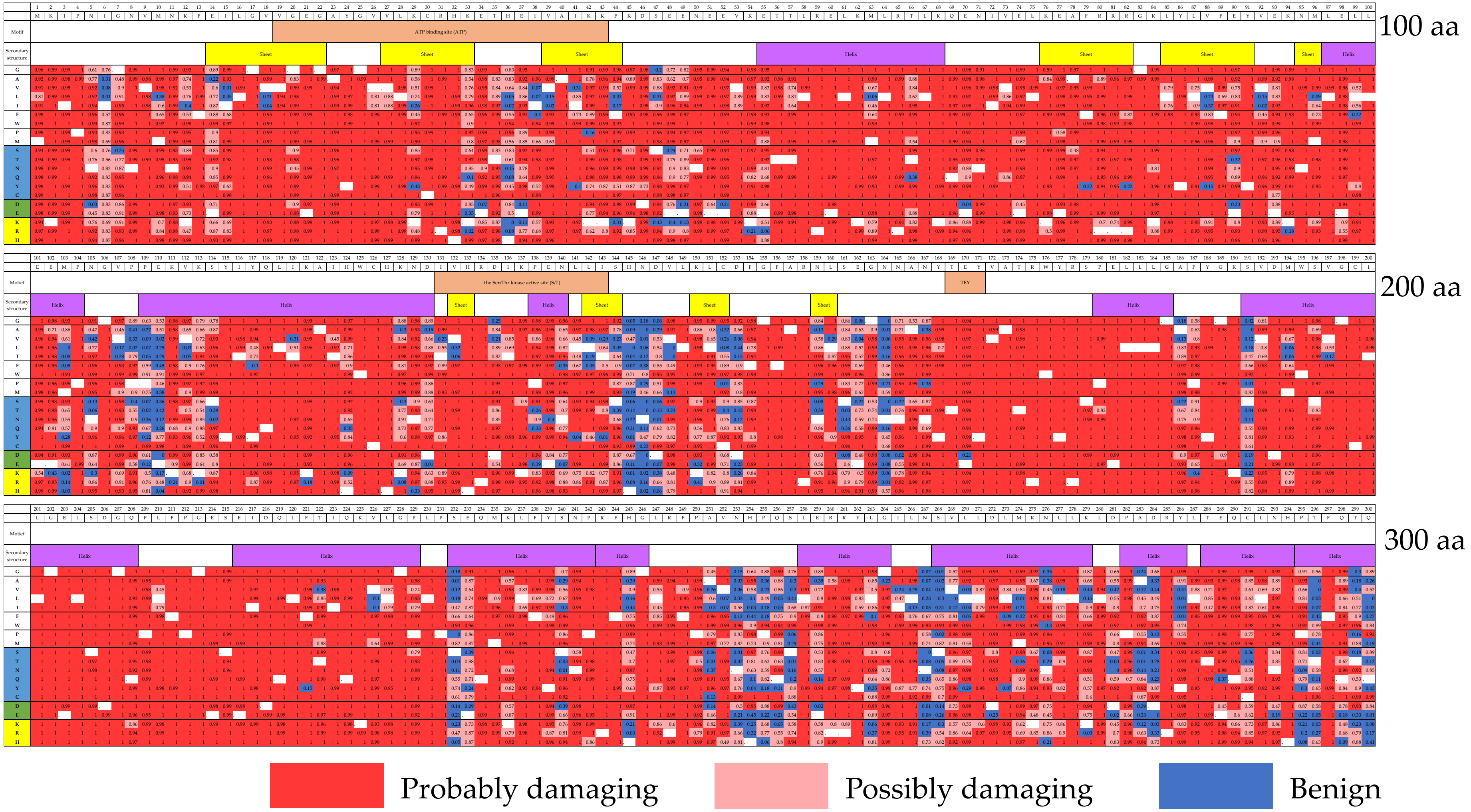

Figure S2. PolyPhen-2 HumVar prediction heatmap. PolyPhen-2 HumVar scores are indicated for each mutation and colored by PolyPhen-2 HumVar prediction; those above 0.447 and 0.909 were highlighted in pink and red, respectively; those below 0.446 were highlighted in blue. Amino acid sequences are shown on the top of the table, where amino acids were categorized as non-polar and polar, neutral (blue), acidic (green), and basic (yellow). WT amino acids such as M1M, K2K, I3I, …was indicated "." and colored in white. The mutation score (0.000~1.000) is within the smallest square. aa; amino acids

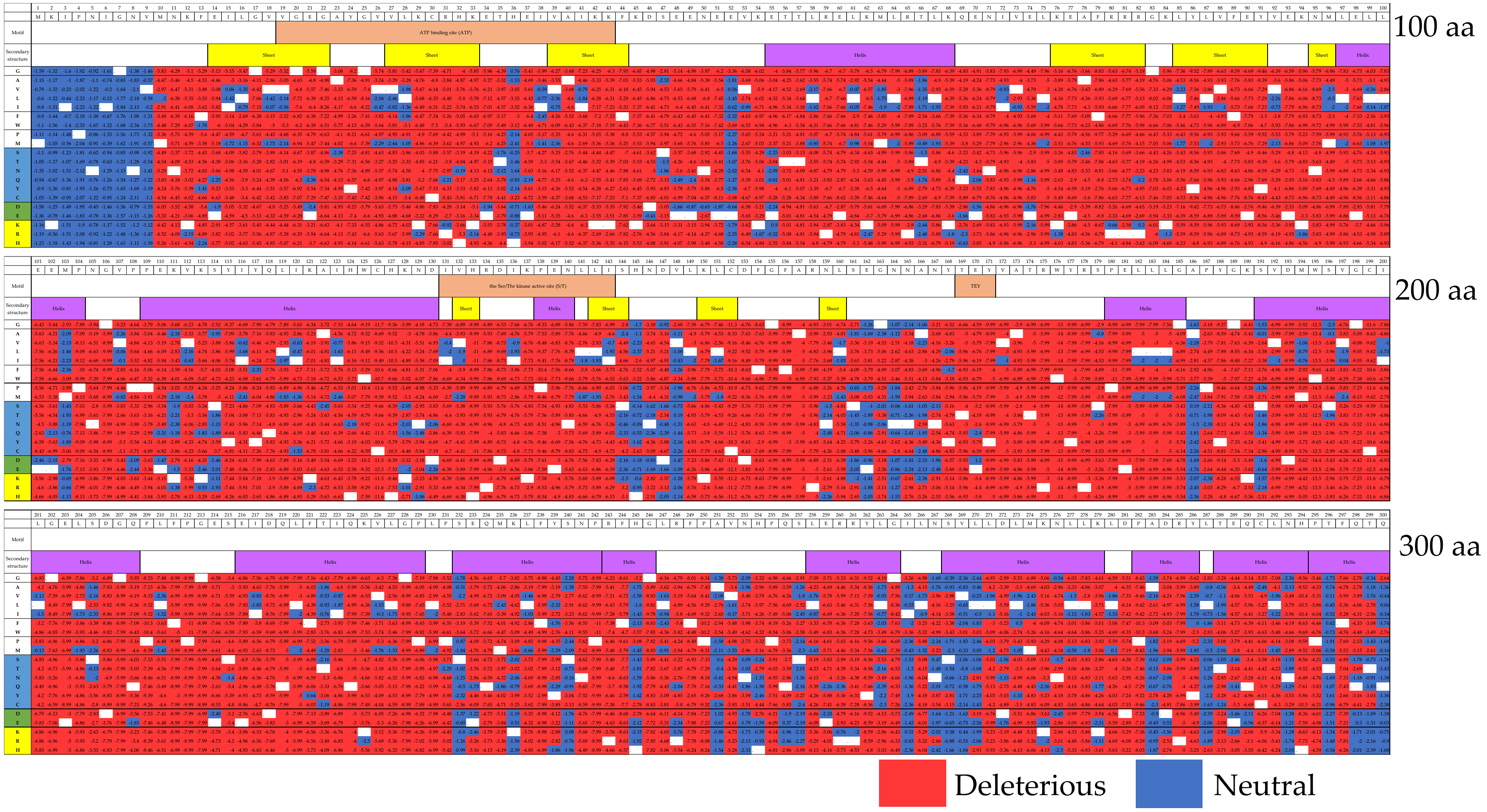

Figure S3. PROVEAN prediction heatmap. PROVEAN scores are indicated for each mutation and colored by prediction; those below -2.5 were highlighted in red and those above were highlighted in blue. Amino acid sequences are shown on the top of the table, where amino acids were categorized as non-polar and polar, neutral (blue), acidic (green), and basic (yellow). WT amino acids such as M1M, K2K, I3I, …was indicated "." and colored in white. The mutation score (-14.986~2.006) is within the smallest square. aa; amino aids

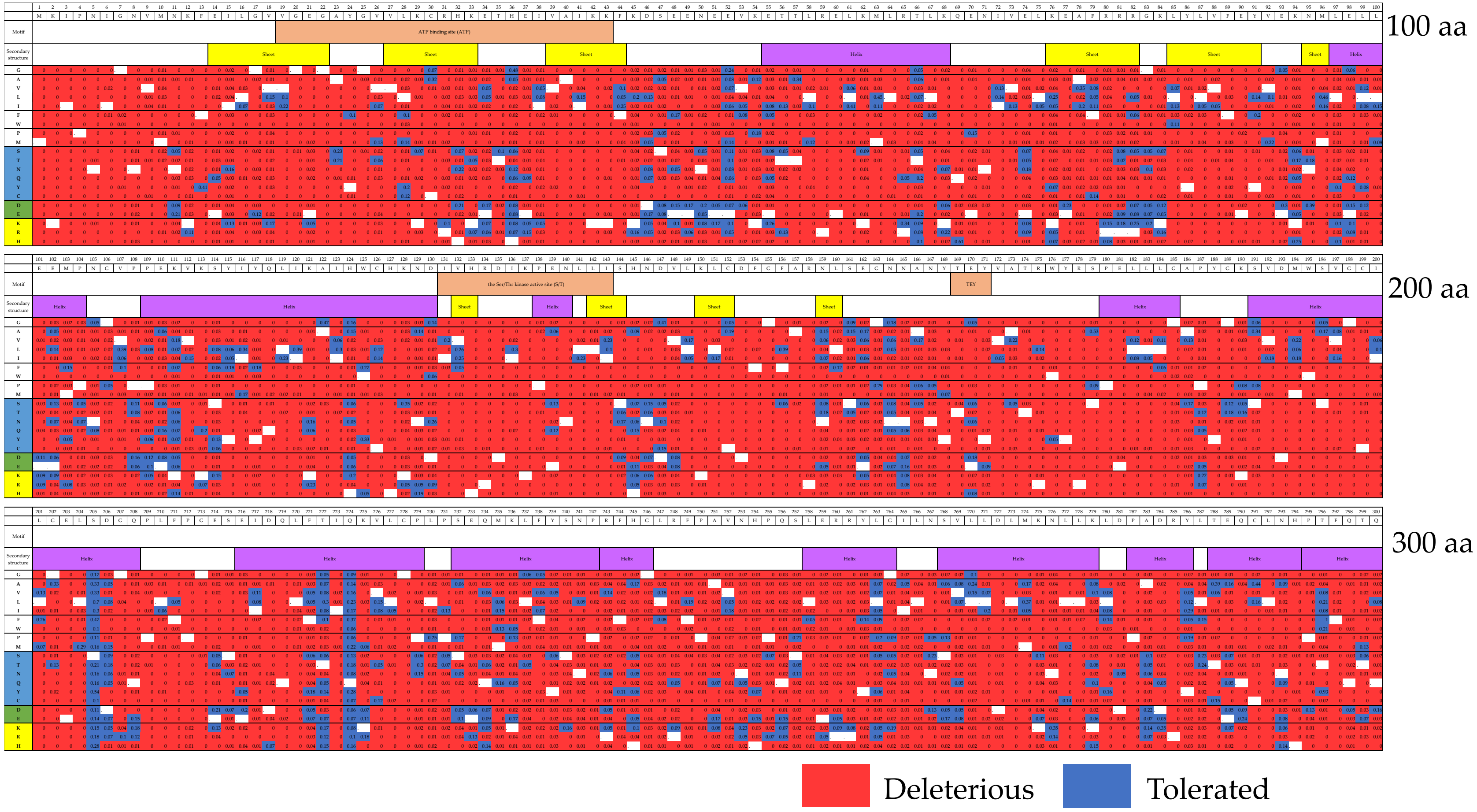

Figure S4. SIFT prediction heatmap. SIFT scores are indicated for each mutation and colored by prediction; those above 0.05 were highlighted in red and considered as "deleterious" mutations, and those below 0.05 were highlighted in blue and considered as "tolerated" mutations. Amino acid sequences are shown on the top of the table, where amino acids were categorized as non-polar and polar, neutral (blue), acidic (green), and basic (yellow). WT such as M1M, K2K, I3I, …was indicated "." and colored in white. WT such as M1M, K2K, I3I, …was indicated "." and colored in white. The mutation score (0.00~1.00) is within the smallest square. Figure S3. PROVEAN prediction heatmap. PROVEAN scores are indicated for each mutation and colored by prediction; those below -2.5 were highlighted in red and those above were highlighted in blue. Amino acid sequences are shown on the top of the table, where amino acids were categorized as non-polar and polar, neutral (blue), acidic (green), and basic (yellow). WT amino acids such as M1M, K2K, I3I, …was indicated "." and colored in white. The mutation score (-14.986~2.006) is within the smallest square. aa; amino acid
